# Computer Vision-Based Optical Odometry Sensors: A Comparative Study of Classical Tracking Methods for Non-Contact Surface Measurement

**DOI:** 10.3390/s25196051

**Published:** 2025-10-01

**Authors:** Ignas Andrijauskas, Marius Šumanas, Andrius Dzedzickis, Wojciech Tanaś, Vytautas Bučinskas

**Affiliations:** 1 Department of Mechatronics, Robotics and Digital Manufacturing, Faculty of Mechanics, Vilnius Gediminas Technical University (VILNIUS TECH), 10223 Vilnius, Lithuania; ignas.andrijauskas@vilniustech.lt (I.A.); marius.sumanas@vilniustech.lt (M.Š.); andrius.dzedzickis@vilniustech.lt (A.D.); 2 Faculty of Engineering Production, University of Life Sciences, 20-618 Lublin, Poland; wojciech.tanas@up.lublin.pl

**Keywords:** computer vision, optical odometry sensors, phase correlation, template matching, optical flow, motion tracking, precision measurement, non-contact sensing

## Abstract

This article presents a principled framework for selecting and tuning classical computer vision algorithms in the context of optical displacement sensing. By isolating key factors that affect algorithm behavior—such as feed window size and motion step size—the study seeks to move beyond intuition-based practices and provide rigorous, repeatable performance evaluations. Computer vision-based optical odometry sensors offer non-contact, high-precision measurement capabilities essential for modern metrology and robotics applications. This paper presents a systematic comparative analysis of three classical tracking algorithms—phase correlation, template matching, and optical flow—for 2D surface displacement measurement using synthetic image sequences with subpixel-accurate ground truth. A virtual camera system generates controlled test conditions using a multi-circle trajectory pattern, enabling systematic evaluation of tracking performance using 400 × 400 and 200 × 200 pixel feed windows. The systematic characterization enables informed algorithm selection based on specific application requirements rather than empirical trial-and-error approaches.

## 1. Introduction

Computer–vision-based optical-displacement sensors have become indispensable in precision metrology, robotics and experimental mechanics, enabling non-contact measurement with sub-pixel spatial resolution and sub-millisecond temporal resolution [1,2]. Their impact spans length-scales from composite-ply monitoring in aerospace structures [3] to whole-plant biomechanics [4]. These systems record a sequence of high-contrast images of a textured surface and infer planar motion by analysing the intensity correspondence between successive frames.

Reliable tracking demands a statistically unique surface pattern. Random speckle fields remain the de-facto standard, with practical guidelines for paint selection and spraying procedures well documented [5]. Targeted image pre-processing—e.g., band-pass filtering and adaptive histogram equalisation—can sharpen correlation peaks on otherwise feature-poor surfaces [6]. Despite the variety of available algorithms, engineers still choose phase correlation, template matching or optical flow largely by trial and error. A recent survey of error-quantification methods for DIC particularly examines statistical error estimation and integration with electron and atomic force microscopy—yet they note that systematic benchmarking across parameter ranges typical in microscopic imaging remains insufficient [7].

While prior studies have focused either on algorithmic refinements or on specific experimental conditions, there remains no unified assessment of how tracker performance degrades or stabilises across textures, window sizes, and displacement regimes. In particular, no quantitative framework exists that allows engineers to move beyond ad-hoc trial-and-error and make informed, texture-dependent choices.

To fill that gap, we conduct a simulation study with 200 × 200 px and 400 × 400 px feed windows that track a multi-circle trajectory over both macro-textured and micro-textured surfaces. A parametric sweep varies the inter-frame step size from 2% to 30% of the window dimension and exposes strong, texture- and scale-dependent regimes. With 400 × 400 windows, phase correlation is unreliable at very small steps (2–6%) but becomes consistently accurate from 8% to 30% (often ≤ 2–3 px), whereas template matching exhibits narrow, texture-specific optima—16%, 22%, and 24% on macro-textures and a single optimum near 6% on micro-textures—and fails on micro-textures when constrained to 200 × 200 windows. Gradient-based optical flow proves viable only at minimal displacements on macro-textures and is unsuitable for precision sensing on micro-textures and for steps above about 6–8%. By mapping these failure thresholds and optimal bands as a function of texture class and window size, we replace ad-hoc tracker choice with a quantitative, texture-first selection framework.

The rest of this paper presents (i) the virtual-camera framework and ground-truth image generator, (ii) detailed error statistics and failure-rate maps across the step-size continuum for both textures and window sizes, and (iii) practical guidelines—expressed as a texture-first decision chart—for choosing and tuning a tracker under typical microscopic-metrology constraints.

## 2. State of the Art

This section briefly surveys the principal approaches now used for vision-based planar odometry: classical trackers (phase correlation, template matching, optical flow), advances in speckle-pattern design, and recent deep-learning alternatives.

### 2.1. Phase Correlation

The seminal phase-correlation method registers two images in the Fourier domain by locating the peak of the inverse cross-power spectrum [8,9]. Using only the phase makes the technique largely immune to uniform illumination changes and additive noise [10]. Sub-pixel precision is commonly obtained by interpolating or up-sampling around the peak [11]; the efficient Fourier-domain refinement proposed by Guizar-Sicairos et al. achieves 1/100-pixel accuracy with only a 16×16 neighbourhood FFT [12]. Padfield introduced masked phase correlation to handle occlusions [13], while pyramid search [14,15] and GPU acceleration [16,17] extend the method to large translations in real time. Blur-robust variants that employ even-powers of the phase spectrum help mitigate peak broadening under defocus blur, by exploiting invariance to centrally symmetric smoothing [18]. The recently proposed differentiable phase-correlation network DPCN++ embeds a learnable feature extractor in a Fourier pipeline and reports state-of-the-art unsupervised registration on heterogeneous data [19].

### 2.2. Template Matching

Template matching slides a reference patch over the next frame and selects the displacement that maximises a similarity metric, typically normalised cross-correlation (NCC) [20]. Least-squares NCC reformulates the optimisation to obtain a closed-form solution that is robust to local intensity variation [21], while FPGA implementations achieve thousand-fold speed-ups for real-time inspection tasks [22]. Quality-aware deep modules such as QATM boost robustness on cluttered scenes [23], and shape-biased CNN features reduce drift under appearance change [24]. A scale-adaptive NCC variant has proved effective for printed-circuit inspection where defects occur at multiple magnifications [25]. Nevertheless, the classic template-update problem—incremental drift due to sequential template replacement—remains a challenge [26,27].

### 2.3. Optical Flow

Optical-flow methods estimate a dense per-pixel motion field under a brightness-constancy assumption. The classical Horn–Schunck formulation further imposes a global spatial smoothness regularizer [28], whereas Lucas–Kanade assumes locally constant (translational) motion within a small neighborhood, with common extensions parameterizing the local warp by an affine model [29]. Modern CNN and transformer variants have surpassed classical methods on public benchmarks. High-resolution one-dimensional attention recovers fine detail at modest cost [30]; GMFlow casts flow as global feature matching [31]; and FlowFormer++ [32] combines masked cost volumes with a transformer decoder. Segment–Anything-guided SAMFlow improves object-boundary coherence [33], whereas kHz-rate optical flow has been demonstrated by dedicated high-speed vision systems [34]. A comprehensive database shows that even the best models struggle on low-texture regions and violate physical constraints without explicit regularisation [35].

### 2.4. Surface Texture and Patterning

Pattern quality governs the DIC correlation peak and convergence; optimization-based speckle design yields high-contrast, isotropic patterns with improved accuracy/efficiency [36,37]. Objective quality metrics based on mean-bias error [38] and defocus tolerance [39] enable pre-test pattern evaluation. CNN-based assessors capture subtler statistical cues than hand-crafted metrics, yielding higher correlation with true DIC error [40,41]. Regular grids or dot arrays facilitate phase-based retrieval but risk one-period mis-registration; adding slight aperiodicity resolves that ambiguity [42]. Under severe motion blur, overlaying a faint grid on a speckle background boosts phase-correlation SNR without sacrificing uniqueness [6].

### 2.5. Deep-Learning Approaches

End-to-end CNN optical-flow networks—FlowNet [43], PWC-Net [44], and RAFT [45]—dominate generic benchmarks, but require domain-specific fine-tuning for microscopic imagery. DeepDIC replaces classical subset correlation with two neural networks that directly predict displacement and strain [46], while pure-CNN DIC approaches have demonstrated millisecond-level (real-time) inference [47]. Embedding physics—such as equilibrium and boundary conditions—into the training objective improves accuracy [48]. Physics-informed neural networks extend this concept to large-deformation measurement [49], and 2D feature-fusion modules enhance illumination invariance in hybrid DIC systems [50]. Even so, classical algorithms remain indispensable in precision metrology because their deterministic error bounds are easier to certify for traceable measurements.

### 2.6. Taxonomy and Comparative Summary

The surveyed approaches can be organised into a taxonomy that separates classical correlation methods (phase correlation, template matching, optical flow), engineered surface patterning, and deep-learning–based models. Each category targets different application domains and exhibits characteristic advantages and limitations. Table 1 summarises their principal use cases, strengths, and weaknesses.

## 3. Materials and Methods

### 3.1. Experimental Design Overview

This study compares the performance of three classical computer-vision methods—phase correlation, template matching, and optical flow—for tracking planar surface displacement under controlled conditions. The experimental approach assumes a fixed 2D planar surface undergoing pure translation, eliminating complications from rotation, scaling, or out-of-plane motion. This simplification is intentional, as it enables a clear, baseline comparison of methods for quicker sensor design without confounding effects from additional degrees of freedom. We acknowledge that real-world scenarios may involve more complex motion; however, translation-only benchmarking provides a necessary first step before extending to rotation, scaling, or perspective distortion. To support reproducibility, the datasets and simulation code are available from the authors upon request via email.

### 3.2. Test Image Sources

The tracking algorithms are evaluated using two distinct test images, each representing a different scale and surface type:

- Macro-scale image: A single image of a randomly textured surface captured using a standard camera setup. This image contains broad, non-repetitive features and moderate lighting variations, simulating typical visual conditions in everyday applications.
- Micro-scale image: A single high-resolution microscopic image of a metallic surface with a random microstructure. The fine-grained texture, combined with inherent sensor noise and illumination inconsistencies, poses a greater challenge to tracking algorithms under high magnification.

Using both macro and micro images of different random-pattern surfaces—one general and one metallic—allows for a robust evaluation of tracking performance across scales and material types. These two cases were deliberately chosen to represent generalized, non-predefined textures typical of CNC-machined parts and laboratory specimens, where the surface pattern is not engineered but arises naturally from the material or imaging setup. As such, they provide a practical and representative basis for benchmarking algorithm robustness in precision metrology contexts.

### 3.3. Subpixel Feed Generation

A critical aspect of this methodology is the implementation of subpixel-accurate feed generation when simulating camera motion. Rather than simple integer-pixel cropping, which would introduce artificial quantisation effects, subpixel resampling is performed to extract image windows at fractional pixel coordinates. In practice, a new window is resampled for each frame along the prescribed trajectory, ensuring that the generated sequence emulates a continuous camera feed. This avoids the staircase artefacts that would arise from integer-pixel cropping, which cannot reproduce the smooth displacements observed in real-world imaging systems.

Conditions. In practice, feed generation starts from a static high-resolution reference image. Camera motion is simulated by extracting a smaller 200×200 or 400×400 pixel window that moves along a prescribed trajectory. To mimic the behaviour of a physical low-resolution sensor, each frame is created by resampling the reference image at fractional offsets rather than by integer-pixel cropping. Importantly, this resampling is performed once for the entire sequence, and the resulting feed is then supplied identically to all algorithms. This ensures that every method operates on the same interpolated data, so performance differences can be attributed solely to algorithmic behaviour rather than input variability.

Interpolation schemes. In this work, bilinear interpolation was selected as the default resampling method, striking a balance between computational efficiency and accuracy. Bilinear interpolation provides continuous intensity variation and avoids discontinuities at integer-pixel boundaries, making it well suited for large parametric studies. Alternative interpolators are possible: bicubic or Lanczos filters yield smoother gradients and higher-frequency fidelity, while B-spline interpolation can further minimise artefacts in high-precision metrology. The chosen bilinear method ensures reproducible and deterministic feed generation at modest computational cost.

**Figure 1:**
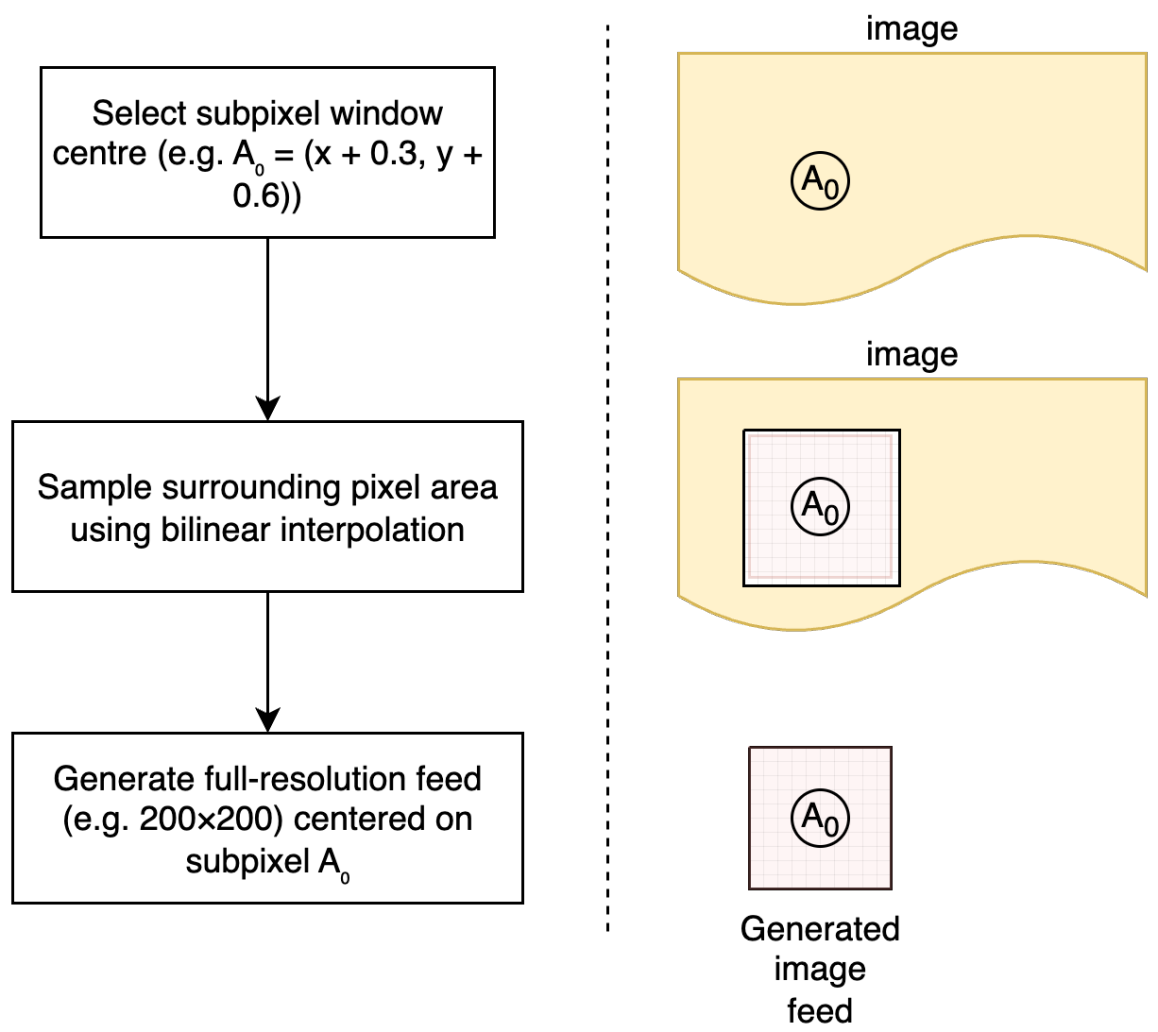
Subpixel feed generation process. The method selects a subpixel window centre (e.g., $A_{0}=\left(x+0.3,y+0.6\right)$), samples the surrounding pixel area using bilinear interpolation, and generates a full-resolution feed centred on the subpixel location.

The subpixel resampling process, illustrated in Figure 1, involves the following:

1. Calculating the desired window centre at fractional coordinates $\left(x_{c}+\Delta x,y_{c}+\Delta y\right)$, where Δx and Δy may be non-integer values;
2. Sampling the source image at the required subpixel locations using bilinear interpolation;
3. Generating a new image feed of specified dimensions centred on the interpolated coordinates.

This technique ensures that displacement estimation errors arise from algorithmic limitations rather than from discretisation artefacts in the test data generation process.

### 3.4. Experimental Parameters

The performance evaluation focuses on two primary experimental variables that directly influence tracking algorithm behavior:

#### 3.4.1. Feed Window Size

The dimensions of the extracted image region (feed window) directly influence tracking performance. Larger windows provide more texture information for correlation but may include irrelevant features, while smaller windows reduce computational cost but may lack sufficient texture for reliable matching.

Square windows were specifically chosen to ensure isotropic tracking performance, as the displacement direction is unknown a priori in general optical sensing applications. Using equal dimensions in both x and y directions eliminates any directional bias that could arise from rectangular windows, ensuring consistent tracking accuracy regardless of motion orientation. This design choice is particularly critical for applications involving arbitrary or circular motion patterns, as demonstrated in our three-circle trajectory experiments.

The selection of 200×200 and 400×400 pixel window sizes represents a careful balance of multiple considerations. The 200×200 pixel compact window provides a 4× computational advantage while still capturing sufficient texture information for correlation-based methods—empirical testing showed this to be near the minimum viable size for reliable tracking on textured surfaces. The 400×400 pixel standard window was chosen as it provides robust texture coverage while remaining computationally tractable for real-time applications on modern hardware, and represents a common sensor resolution in industrial vision systems. The 2× scaling factor between windows allows direct assessment of quadratic computational scaling effects while maintaining comparable texture statistics.

The initial demonstration experiment employs the 400×400 pixel window to illustrate fundamental performance characteristics and tracking behavior for all three methods under controlled conditions. The parametric analysis then systematically compares both window sizes, enabling quantitative assessment of the trade-offs between computational efficiency and tracking reliability across the full parameter range.

#### 3.4.2. Step Size

The magnitude of displacement between consecutive frames, expressed as a percentage of the feed window dimension, determines the tracking difficulty. Percentage-based metrics ensure scale-invariant performance characterization, enabling direct comparison across different window sizes and generalization to various sensor resolutions—a standard approach in computer vision literature for motion characterization.

**Figure 2:**
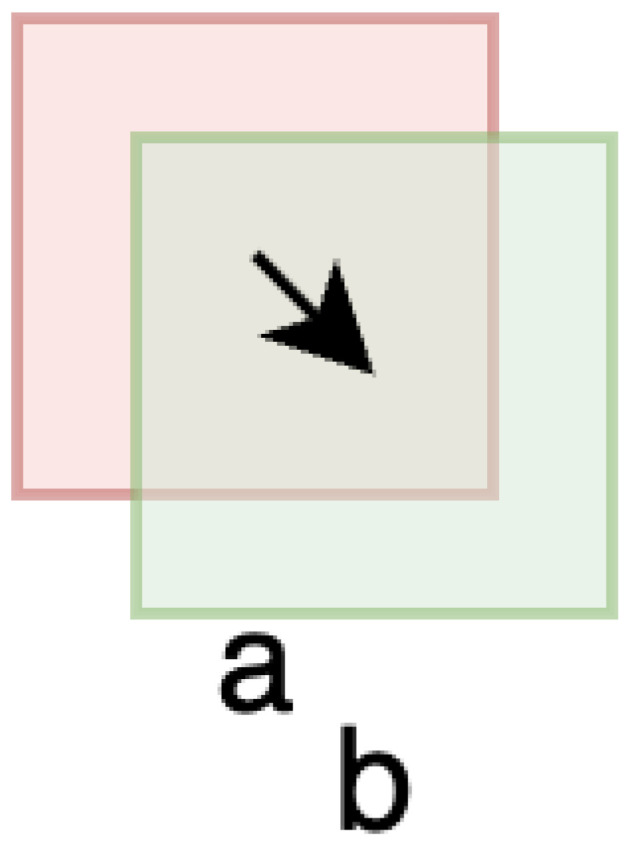
Illustration of the step size concept: windows **a** and **b** represent consecutive feed windows at different timesteps. The arrow indicates the direction of camera (or feed) movement. Window displacement introduces new image content while maintaining partial overlap, enabling correlation-based tracking.

For example, consider a simplified 4 × 4 pixel feed window (window ‘a’ in Figure 2) that displaces by 1 pixel magnitude in the southeast direction. This results in approximately $\sqrt{2}$ pixel movement in both x and y directions (1 pixels each), introducing 7 new pixels out of 16 total pixels (43.75% new information), while maintaining 9 pixels of overlap. The step size of 35.35% (1.41 pixel displacement/4 pixel window dimension) demonstrates how even modest displacements can introduce substantial new visual information that challenges tracking algorithms.

The 2–30% range encompasses the full spectrum of practical tracking scenarios. The lower bound of 2% (8 pixels for a 400-pixel window) approaches the minimal detectable motion where displacement becomes comparable to sensor noise, testing algorithms’ ability to distinguish genuine motion from measurement uncertainty. The upper bound of 30% represents the practical limit for correlation-based methods as less than 50% frame overlap typically causes tracking failure due to insufficient common features. Small step sizes (2–5%) represent high-precision positioning applications, medium sizes (10–20%) correspond to standard industrial vision tasks, while large sizes (20–30%) simulate challenging rapid motion or reduced frame rate conditions.

This parameter directly affects the amount of new visual information introduced per frame and challenges the fundamental assumptions of correlation-based tracking methods. The parametric study systematically varies step sizes from 2% to 30% in 2% increments, providing sufficient resolution to identify performance transitions and optimal operating regions for each algorithm.

#### 3.4.3. Controlled Conditions and Reproducibility

Both parameters were strictly controlled across all tested algorithms to ensure comparability. Window sizes and step increments were fixed a priori, and identical image regions were supplied to each method under the same motion trajectories. This guarantees that performance differences arise solely from algorithmic behavior rather than input variability, thereby ensuring reproducibility of results. The controlled parameter set is summarised in Table 2.

### 3.5. Performance Metrics

Algorithm performance is quantified through several error measures comparing the estimated displacement path to the known ground truth:

- Mean absolute error: Average Euclidean distance between estimated and true positions across all frames;
- Maximum error: Peak deviation magnitude, indicating worst-case performance;
- Standard deviation of error: Measure of tracking consistency and stability;
- Final position error: Accumulated drift after complete path traversal;
- Error evolution: Temporal analysis of how tracking accuracy degrades or stabilises over long sequences.

### 3.6. Path Generation

**Figure 3:**
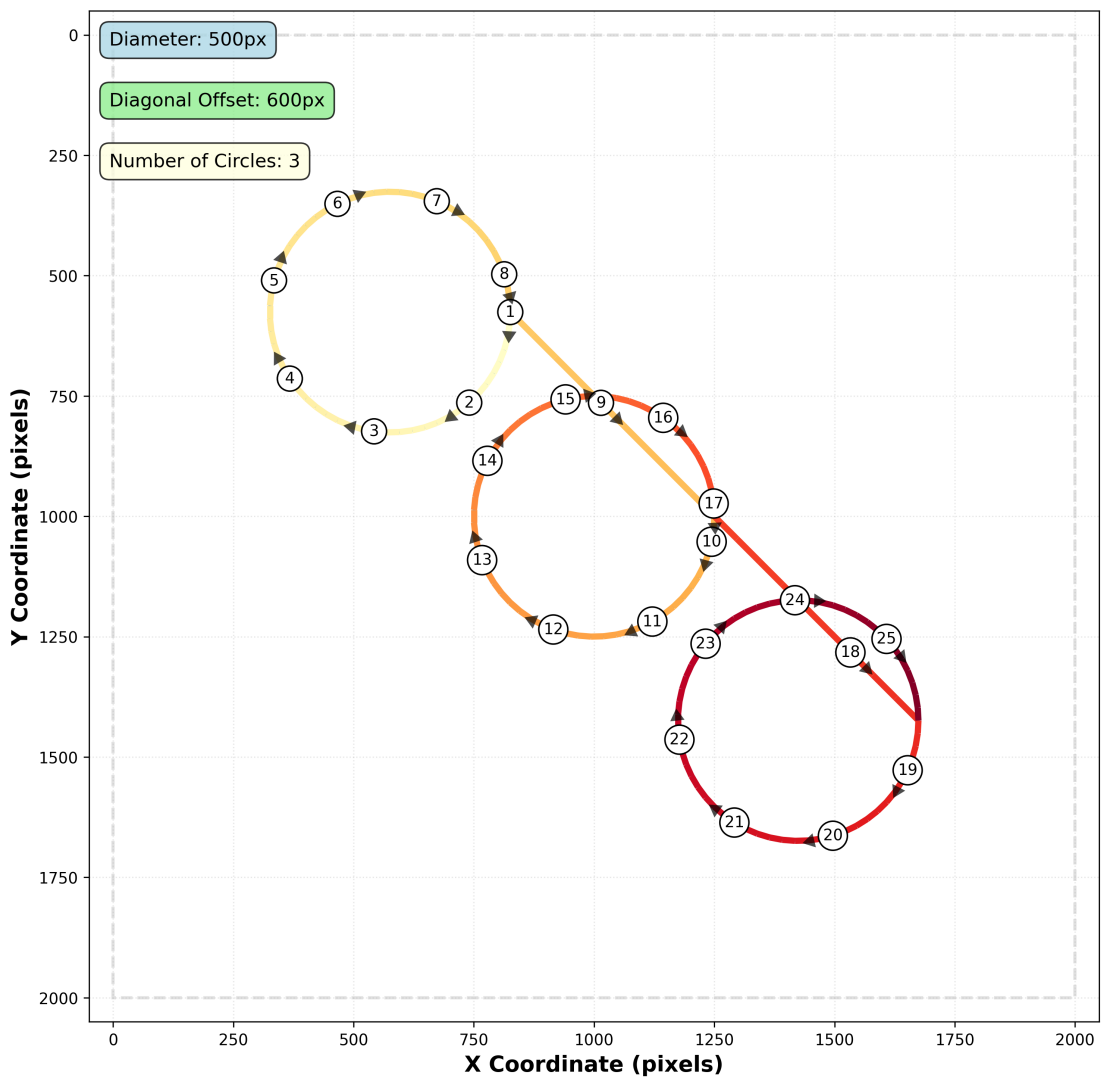
Multi-circle path generation pattern showing three circular segments with diagonal transitions. The test trajectory consists of 500-pixel diameter circles connected by 600-pixel diagonal offsets, combining curved and linear motion segments for comprehensive algorithm evaluation. The path fades progressively from yellow (start) to dark red (end), helping to intuitively visualize motion progression. Direction is further emphasized using arrows placed at regular intervals. Numbered circles indicate arrow positions and are used purely for visual clarity—they do not correspond to exact temporal or spatial steps. The y-axis follows the standard image coordinate convention with origin (0,0) at the top-left corner, as used by OpenCV.

The test trajectory consists of a continuous path traversing multiple circular segments connected by diagonal transitions, as illustrated in Figure 3. This path design provides both curved motion for testing temporal consistency and straight-line segments for evaluating directional accuracy.

The path is generated as a sequence of three circular loops, each with a diameter of 500 pixels, positioned along a diagonal offset of 600 pixels. The circles are arranged in a northwest-to-southeast progression, with each subsequent circle center displaced by the diagonal offset distance at a 45-degree angle.

The path traversal follows a specific sequence to ensure consistent motion characteristics. Beginning at the rightmost point of the first circle (designated as 1), the trajectory proceeds clockwise around the complete circumference. Upon completing the first circle, the path transitions diagonally from the end point to the rightmost coordinate of the second circle, maintaining the northwest-to-southeast progression. This pattern continues for all subsequent circles, with each circular segment followed by a diagonal transition to the next circle’s starting position.

This configuration ensures that the tracking algorithms encounter varied motion patterns while maintaining sufficient texture overlap between consecutive frames. Each circular segment is parametrised to maintain constant angular velocity, with the number of discrete points determined by the specified step size to ensure uniform spatial sampling. Smooth transitions between circles are achieved through linear interpolation along the diagonal connecting segments, preventing abrupt direction changes that could introduce tracking discontinuities.

The complete path length significantly exceeds the camera feed window dimensions, enabling evaluation of long-term tracking stability and cumulative error accumulation over extended motion sequences. This multi-circle trajectory effectively exercises the algorithms’ ability to maintain accuracy across diverse motion patterns while providing a deterministic ground truth for quantitative performance assessment.

### 3.7. Implementation Details

All three tracking methods are implemented using consistent preprocessing steps:

- Gaussian blur for noise reduction (kernel size: 3×3, σ=1.0);
- Contrast-limited adaptive histogram equalisation (CLAHE) for illumination normalisation;
- Hanning windowing for phase correlation to reduce edge effects.

The experimental framework captures both the estimated trajectory and associated confidence metrics for each method, enabling comprehensive comparison of accuracy, robustness, and computational characteristics under varying conditions.

## 4. Results

### 4.1. Experimental Procedure

The experimental evaluation was conducted using a systematic approach to compare the three classical computer vision methods under controlled conditions. A virtual camera system was implemented to generate synthetic image sequences with precise ground truth trajectories, enabling quantitative assessment of tracking accuracy across diverse surface textures and operational parameters.

**Figure 4:**
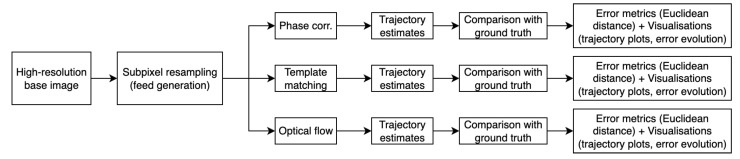
Overview of the test evaluation process. A high-resolution base image is used to generate subpixel-shifted feeds, which are processed by the three tracking algorithms. Their estimated trajectories are compared with the ground truth path, and accuracy is quantified using Euclidean distance errors and visualised through trajectory plots and error evolution curves.

Figure 4 illustrates the complete test evaluation workflow, from feed generation to algorithm comparison and error quantification.

#### 4.1.1. Virtual Camera Setup

The experimental framework utilised two distinct surface texture types—macro-textured general and micro-textured metal surfaces—to evaluate algorithm performance across different spatial frequency characteristics. The system employed virtual camera feed windows of two sizes: 200×200 pixels (compact configuration) and 400×400 pixels (standard configuration). Feed generation was performed with subpixel accuracy through bilinear interpolation, simulating realistic camera motion without discretisation artefacts. A step size of 10 pixels was selected for the initial experiments, representing 2.5% and 5% of the standard and compact feed window dimensions respectively, providing moderate motion between consecutive frames while maintaining sufficient texture overlap for correlation-based methods.

#### 4.1.2. Test Trajectory Execution

The predefined three-circle path was executed with each frame position calculated to subpixel precision. To ensure statistical robustness, each experimental configuration was repeated 10 times with randomised starting positions within the texture field. At each trajectory point, the virtual camera generated a new image feed centered on the true position coordinates. The three tracking algorithms—phase correlation, template matching, and optical flow—simultaneously processed consecutive frame pairs to estimate displacement vectors. Each method maintained an independent position estimate, accumulating displacement measurements from the initial starting position.

#### 4.1.3. Performance Assessment

**Figure 5:**
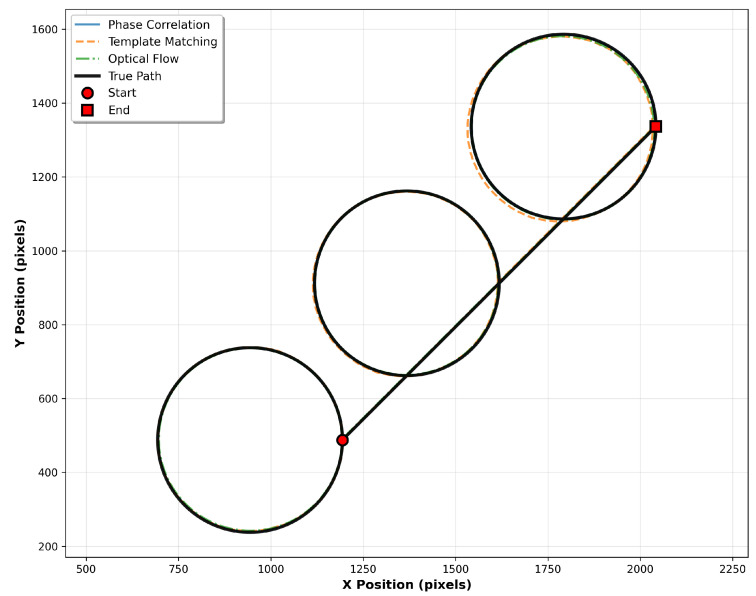
Trajectory comparison showing the true circular path (black) and estimated paths from phase correlation (blue), template matching (orange), and optical flow (green). Start and end points are marked to indicate path direction and cumulative drift effects.

The experimental results were analysed through multiple complementary visualisations and metrics, with all data aggregated across the 10 independent runs to provide statistically meaningful performance characterisation. Figure 5 presents the complete trajectory comparison from a representative single run, showing the ground truth path alongside all three estimated trajectories. The tracking accuracy is clearly visualised, with deviations from the true path indicating algorithm-specific error characteristics.

The Euclidean distance $d_{t}$ between the true position $\left(x_{t}^{\mathrm{true}},y_{t}^{\mathrm{true}}\right)$ and the estimated position $\left(x_{t}^{\mathrm{est}},y_{t}^{\mathrm{est}}\right)$ at step *t* is computed as

(1) $$d_{t}=\sqrt{{\left(x_{t}^{\mathrm{true}}-x_{t}^{\mathrm{est}}\right)}^{2}+{\left(y_{t}^{\mathrm{true}}-y_{t}^{\mathrm{est}}\right)}^{2}}$$

**Figure 6:**
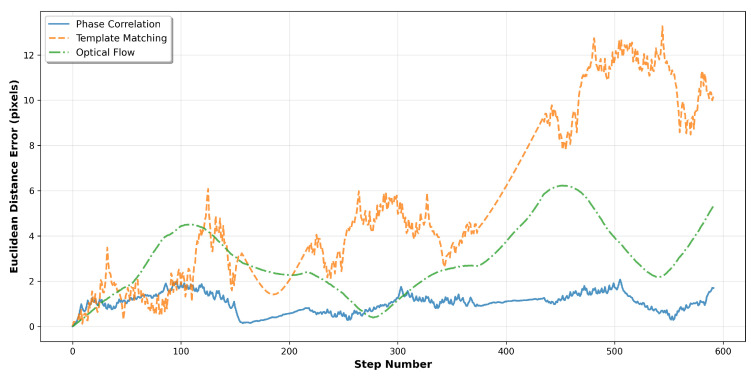
Error evolution over the complete trajectory sequence. The plot shows instantaneous Euclidean distance errors for each tracking method, revealing temporal stability characteristics and cumulative drift behaviour.

The temporal evolution of tracking errors is analysed in Figure 6, which plots the Euclidean distance between true and estimated positions, computed as in Equation (1), as a function of step number. This visualisation reveals how tracking accuracy degrades over time and identifies periods of increased estimation difficulty, such as during transitions between circular segments.

#### 4.1.4. Statistical Error Analysis

**Figure 7:**
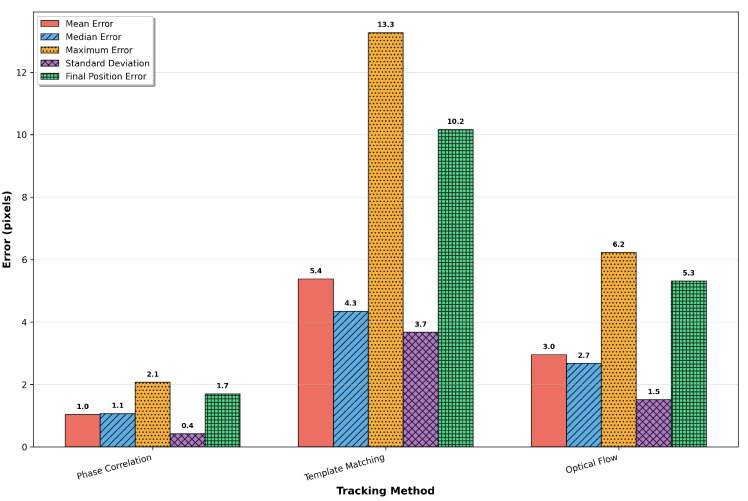
Comprehensive error statistics comparison showing mean, median, maximum, standard deviation, and final position errors for each tracking method. Numerical values are displayed above each bar for precise quantitative assessment.

Comprehensive error statistics are presented in Figure 7, providing quantitative comparison across five key performance metrics. The mean absolute error indicates overall tracking accuracy, while the median error offers a robust measure less sensitive to outliers. Maximum error represents worst-case performance, and standard deviation quantifies tracking consistency. Final position error measures the cumulative drift after traversing the complete path, which is critical for long-term tracking applications.

**Figure 8:**
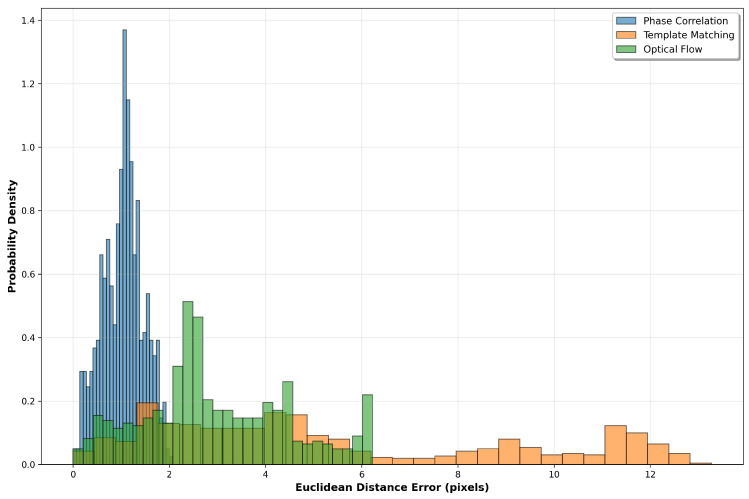
Error distribution histograms showing the probability density of Euclidean distance errors for each tracking method. The shape and spread of distributions provide insights into tracking consistency and outlier behaviour.

The distribution of tracking errors is further examined through probability density analysis (Figure 8), which reveals the statistical characteristics of each method’s performance. Narrow distributions indicate consistent tracking behaviour, while wider distributions or multiple peaks suggest periods of variable accuracy.

#### 4.1.5. Visual Validation

**Figure 9:**
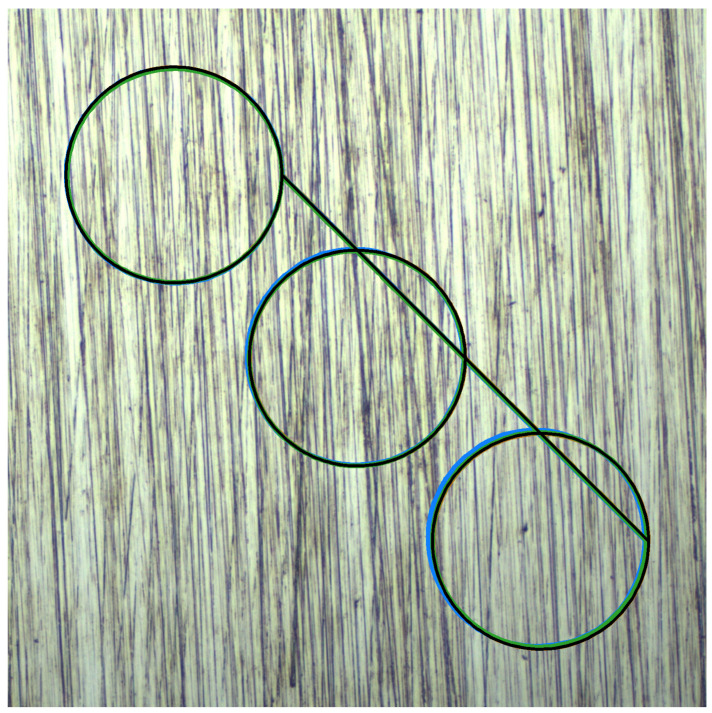
Trajectory overlay on the actual metal surface texture showing the true path (solid black) and estimated paths with distinct line styles: phase correlation (solid blue), template matching (dashed orange), and optical flow (solid green). The cropped view focuses on the tracking region for detailed visual assessment.

Figure 9 provides visual validation by overlaying all estimated trajectories onto the actual surface texture used during tracking. This representation demonstrates how the algorithms perform on realistic surface patterns and shows the spatial relationship between tracking errors and surface features. The image is automatically cropped to focus on the trajectory region, eliminating unused background areas for clearer visualisation.

### 4.2. Parametric Analysis Results

The parametric analysis systematically examined algorithm performance across step sizes ranging from 2% to 30% of the feed window dimension, using two representative window sizes: 200×200 pixels (compact) and 400×400 pixels (standard). This comprehensive evaluation was conducted on both macro-textured and micro-textured surfaces, with each configuration repeated 10 times using randomised starting positions to ensure statistical reliability. The aggregated results reveal distinct sensitivity patterns and operational limits for each tracking method under varying motion and texture conditions.

#### 4.2.1. Macro-Texture Surface Performance

**Figure 10:**
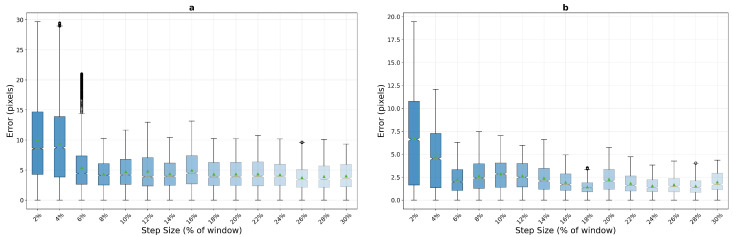
Phase correlation performance analysis on macro-textured surfaces showing error distribution across step sizes for 200×200 pixel (**a**) and 400×400 pixel (**b**) windows. Box plots reveal median error trends and variance characteristics under increasing motion stress, aggregated over 10 runs with random starting positions.

On macro-textured surfaces, phase correlation demonstrates unexpected behaviour patterns that challenge conventional assumptions about frequency-domain tracking methods. As shown in Figure 10, the algorithm exhibits poor performance at very low step sizes but achieves remarkable stability in mid-range motion conditions. For the 400×400 pixel window, phase correlation shows elevated errors at 2% step size (mean error 7.8 pixels) that improve significantly by 8% (3.3 pixels) and reach optimal performance in the 12–16% range (2.7 and 2.0 pixels respectively), maintaining consistent sub-pixel accuracy with low variance (std dev 1.61 pixels at 12%). The compact 200×200 window exhibits even more pronounced low-speed difficulties, with mean errors of 10.1 pixels at 2% and 9.5 pixels at 4% step sizes, but demonstrates stability from 14% onwards (4.4 pixels) through the remainder of the tested range.

This unexpected behavior suggests that phase correlation’s frequency-domain analysis may struggle with minimal displacement scenarios where correlation peaks become ambiguous due to insufficient motion information. The algorithm appears to require sufficient inter-frame displacement to generate reliable frequency-domain signatures for accurate registration.

**Figure 11:**
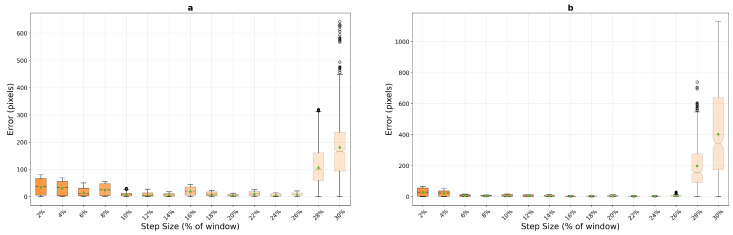
Template matching performance analysis on macro-textured surfaces across window sizes, 200×200 pixels (**a**) and 400×400 pixels (**b**). The method shows selective excellence at specific step sizes with the larger window but exhibits complete performance degradation with the compact configuration.

Template matching exhibits fundamentally different behavior patterns on macro-textured surfaces, as illustrated in Figure 11. The method demonstrates exceptionally poor performance at very low step sizes, with mean errors of 31.8 and 36.3 pixels at 2% step size for the 400×400 and 200×200 window configurations respectively—substantially worse than both phase correlation (7.8 and 10.1 pixels) and optical flow (5.1 pixels for 200 px window). However, template matching achieves remarkable selective excellence at specific step sizes with the larger window, particularly at 16% (2.2 pixels), 22% (2.4 pixels), and 24% (2.8 pixels), where it matches or outperforms all other methods with excellent stability (std dev 1.42 pixels at 16%). The compact window configuration completely eliminates these selective excellence characteristics, with minimum mean error never dropping below 5.4 pixels across all tested step sizes.

**Figure 12:**
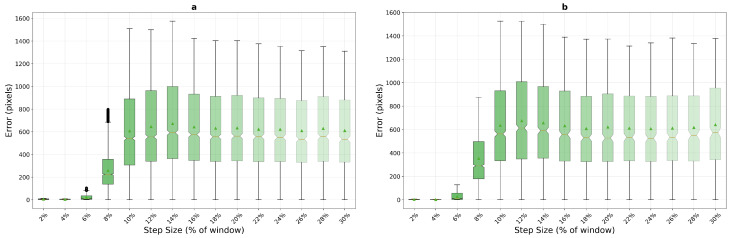
Optical flow performance analysis on macro-textured surfaces, 200×200 pixels (**a**) and 400×400 pixels (**b**), demonstrating superior low-speed tracking but catastrophic failure at moderate to high step sizes. The method shows consistent failure patterns across both window configurations.

Optical flow presents the most distinctive performance profile among the three methods on macro-textured surfaces, as shown in Figure 12. The algorithm demonstrates superior performance at very low step sizes with the compact window, achieving mean errors of 5.1 pixels at 2% and 4.0 pixels at 4% step size for the 200×200 configuration—significantly outperforming both phase correlation (10.1 and 9.5 pixels) and template matching (36.3 and 32.2 pixels) in this critical low-speed regime, albeit with higher variance (std dev 3.16 pixels). However, optical flow exhibits catastrophic failure at 8% step size, with mean errors jumping to 258.8 pixels and stabilizing in a failure mode around 608–610 pixels for step sizes from 10% onwards. This dramatic transition represents a complete breakdown of the brightness constancy assumption underlying the Lucas–Kanade method.

These quantitative results reveal distinct operational domains for each algorithm on macro-textured surfaces. For applications requiring consistent sub-pixel accuracy across a broad range of motion speeds, phase correlation emerges as the most reliable choice, particularly with the 400×400 pixel window where it maintains mean errors below 3.5 pixels for all step sizes from 8% onwards. Template matching, despite its poor low-speed performance, offers the highest absolute accuracy at specific step sizes (2.2 pixels at 16%) when motion parameters can be controlled. Optical flow, while providing the best low-speed tracking for compact windows, should be restricted to applications where inter-frame displacement never exceeds 6% of the window dimension to avoid catastrophic failure.

#### 4.2.2. Micro-Texture Surface Performance

**Figure 13:**
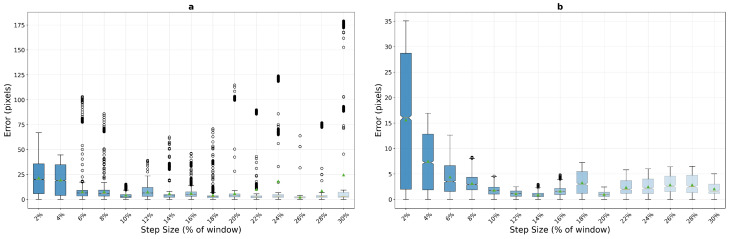
Phase correlation performance on micro-textured surfaces, 200×200 pixels (**a**) and 400×400 pixels (**b**), showing enhanced stability at low step sizes due to richer frequency content. The algorithm maintains consistent sub-pixel accuracy across a broader operational range.

The micro-textured surface experiments reveal significantly different algorithm behaviours due to the higher spatial frequency content and finer texture details. Contrary to initial expectations, phase correlation performance on micro-textured surfaces (Figure 13) shows degraded accuracy at low step sizes compared to macro-textured surfaces. For the 400×400 pixel window, mean errors reach 15.8 pixels at 2% step size (compared to 7.8 pixels on macro-texture) and 7.3 pixels at 4%, before achieving excellent sub-pixel performance in the 8–16% range with mean errors of 1.9–2.1 pixels and remarkable stability (std dev 1.05 pixels at 10%). The 200×200 window exhibits even more severe low-speed difficulties with mean errors of 21.6 pixels at 2% (versus 10.1 on macro-texture), stabilizing only from 8% onwards at approximately 7.7 pixels.

**Figure 14:**
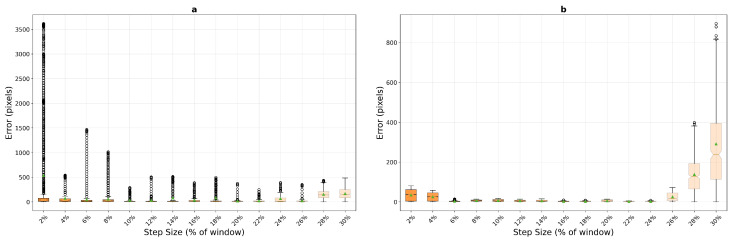
Template matching performance on micro-textured surfaces, 200×200 pixels (**a**) and 400×400 pixels (**b**), exhibiting superior accuracy and consistency compared to macro-textured results. The fine texture features enable robust correlation-based tracking at low to moderate motion levels.

Template matching demonstrates dramatically different and somewhat paradoxical performance characteristics on micro-textured surfaces (Figure 14). With the 400×400 pixel window, the algorithm shows poor initial performance at 2% step size (34.7 pixels, similar to macro-texture’s 31.8 pixels) but achieves a remarkable improvement at 6% step size with mean error dropping to 4.0 pixels—significantly better than its best performance on macro-textured surfaces. However, this excellent performance window is narrow, with errors increasing to 6.0 pixels at 8% step size. The compact 200×200 window configuration exhibits catastrophic failure on micro-textured surfaces, with mean errors of 537.2 pixels at 2% step size—an order of magnitude worse than on macro-textured surfaces (36.3 pixels)—and never achieving mean errors below 19.8 pixels across the entire parameter range. This extreme sensitivity suggests that fine texture details can create severe aliasing or ambiguity problems for correlation-based matching when the search window is insufficiently large.

**Figure 15:**
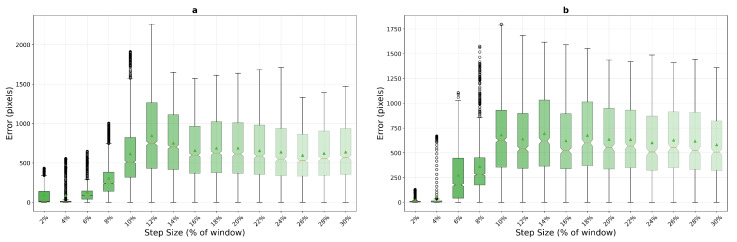
Optical flow performance on micro-textured surfaces, 200×200 pixels (**a**) and 400×400 pixels (**b**), confirming consistent failure patterns regardless of texture characteristics. The algorithm remains viable only for minimal inter-frame displacements.

Optical flow performance on micro-textured surfaces (Figure 15) reveals fundamental limitations of gradient-based methods when applied to high-frequency textures. Unlike its reasonable performance on macro-textured surfaces at low speeds, optical flow exhibits poor accuracy from the outset on micro-textures, with mean errors of 95.9 pixels at 2% step size for the 200×200 window—nearly 20 times worse than on macro-texture (5.1 pixels). The degradation continues progressively through 89.7 pixels at 4% and 129.1 pixels at 6%, reaching complete failure by 8% (307.3 pixels). The 400×400 window configuration shows similarly poor performance with mean errors ranging from 35.7 pixels at 2% to complete tracking failure (856+ pixels) from 10% onwards. These results confirm that the fine spatial details in micro-textured surfaces violate the smoothness assumptions inherent in the Lucas–Kanade optical flow formulation, rendering the method unsuitable for high-frequency texture tracking regardless of motion parameters.

The micro-texture results reveal critical texture-dependent performance characteristics that significantly impact algorithm selection. Phase correlation, despite worse low-speed performance compared to macro-textures, achieves superior steady-state accuracy (1.9 pixels) when sufficient motion exists to generate distinct frequency signatures. Template matching exhibits a narrow window of exceptional performance at 6% step size (4.0 pixels) with the larger window, but suffers from extreme sensitivity to window size and catastrophic failure with compact configurations. The fine texture details that theoretically should aid matching instead create severe challenges at most operating points. Optical flow proves entirely unsuitable for micro-textured surfaces, with the high-frequency content fundamentally incompatible with its mathematical assumptions. For micro-textured surface tracking, phase correlation with a 400×400 pixel window operating at 8–16% step sizes provides the most reliable solution, while template matching can offer superior accuracy if motion parameters can be precisely controlled around its 6% optimal point.

#### 4.2.3. Comparative Performance Trends

**Figure 16:**
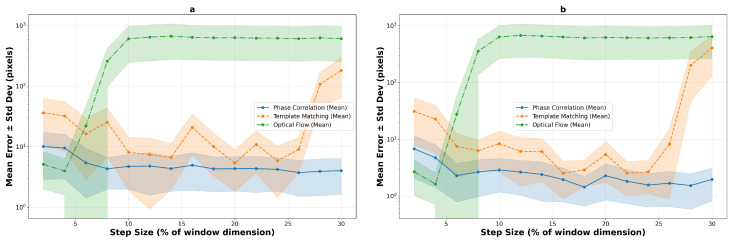
Logarithmic performance trends on macro-textured surfaces showing mean error evolution with step size for 200×200 pixels (**a**) and 400×400 pixels (**b**) windows. Shaded regions indicate standard deviation bounds across 10 runs, revealing algorithm stability characteristics.

**Figure 17:**
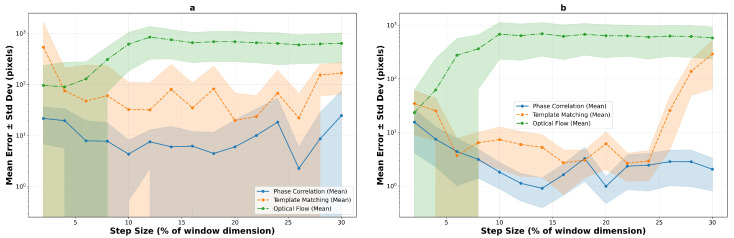
Logarithmic performance trends on micro-textured surfaces, 200×200 pixels (**a**) and 400×400 pixels (**b**), demonstrating improved overall stability for correlation-based methods due to richer texture content. The enhanced spatial frequency characteristics enable more reliable tracking across broader operational ranges.

The logarithmic performance trend analysis reveals fundamental algorithmic differences under increasing motion stress across both texture types. Figure 16 illustrates the performance evolution on macro-textured surfaces, while Figure 17 shows the corresponding trends for micro-textured surfaces. The contrasting behaviors between texture types highlight the critical importance of surface characteristics in algorithm selection.

On macro-textured surfaces, template matching shows relatively high errors at small step sizes (2–8%), improves to mid single-digit errors around 10–20%, and then degrades for larger steps (26–30%). Phase correlation shows moderate initial errors but achieves competitive performance from 8–10% step sizes onwards, with errors near 4–6 pixels for 200×200 windows and 1–3 pixels for 400×400 windows. Optical flow, while demonstrating the best low-speed result with compact windows (5.1 pixels at 2%), becomes unreliable beyond 6–8% step size.

Micro-textured surfaces present a markedly different performance landscape. Phase correlation exhibits significantly elevated errors at low speeds (15.8 pixels at 2% versus 7.8 on macro-texture) but achieves superior steady-state performance with sub-2-pixel accuracy from 10% onwards. Template matching shows extreme variability, with a narrow window of exceptional performance at 6% step size (4.0 pixels) surrounded by regions of poor tracking. Most notably, optical flow demonstrates complete unsuitability for micro-textured surfaces, with errors exceeding 35 pixels even at minimal step sizes, confirming that high-frequency texture content fundamentally violates the algorithm’s smoothness assumptions.

#### 4.2.4. Failure Analysis and Operational Limits

**Figure 18:**
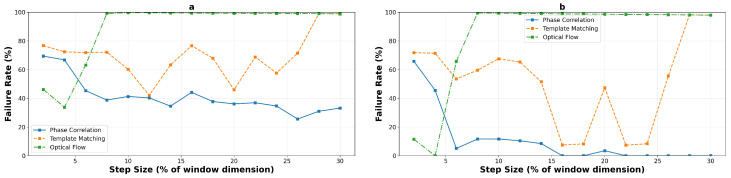
Failure rate analysis on macro-textured surfaces, 200×200 pixels (**a**) and 400×400 pixels (**b**), using 5-pixel error threshold. Charts show percentage of tracking points exceeding acceptable error limits, identifying operational boundaries for reliable performance across 10 independent runs.

**Figure 19:**
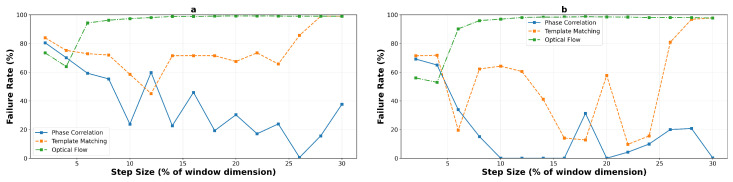
Failure rate analysis on micro-textured surfaces, 200×200 pixels (**a**) and 400×400 pixels (**b**), revealing enhanced reliability for correlation-based methods due to improved texture characteristics. The richer spatial frequency content significantly reduces failure rates at moderate step sizes.

Using a 5-pixel error threshold for precision applications, the failure rate analysis provides critical insights into operational reliability. Figure 18 and Figure 19 present the detailed failure characteristics for macro- and micro-textured surfaces respectively, aggregated across all experimental runs. For a concise overview of the reliable operating zones for each algorithm, texture type, and window size, Table 3 summarizes the identified boundaries.

On macro-textured surfaces, phase correlation demonstrates high failure rates at very low step sizes but achieves excellent reliability beyond specific thresholds: from 8% onwards for the 400×400 window (mean error dropping from 7.8 to 3.3 pixels), and from 14% onwards for the 200×200 window. Template matching exhibits binary behavior—either failing completely or achieving sub-threshold performance at its selective excellence points (16%, 22%, and 24% for the large window). Optical flow maintains reliability only below 6% step size before experiencing catastrophic and permanent failure.

Micro-textured surfaces present more challenging operational constraints. Phase correlation requires higher step sizes to achieve reliability, with the 400×400 window configuration becoming reliable only from 8% onwards (transitioning from 7.3 to 3.0 pixels mean error). Template matching achieves sub-threshold performance only in a narrow window around 6% step size (4.0 pixels mean error), with failure at all other operating points. Most critically, optical flow never achieves reliable performance on micro-textured surfaces, with all tested configurations exceeding the 5-pixel threshold even at minimal step sizes, confirming its fundamental unsuitability for high-frequency texture tracking.

## 5. Discussion

The systematic experimental evaluation across macro and micro-textured surfaces reveals complex, texture-dependent performance characteristics that fundamentally challenge conventional assumptions about tracking algorithm selection for optical displacement sensing applications. The comprehensive analysis, validated through multiple runs with randomized starting positions, provides unprecedented insights into the interplay between surface texture, window size, motion magnitude, and tracking reliability.

### 5.1. Texture-Dependent Algorithm Behavior

The most striking finding is the profound impact of surface texture characteristics on algorithm performance. Phase correlation, traditionally considered a robust frequency-domain method, exhibits dramatically different behavior patterns between macro and micro-textured surfaces. On macro-textures, the algorithm shows poor performance at low step sizes (7.8 pixels at 2% for 400 px window) but achieves excellent stability from 8% onwards (3.3 pixels). However, on micro-textures, this low-speed degradation is significantly amplified (15.8 pixels at 2%), suggesting that high-frequency texture content creates ambiguous frequency-domain signatures at minimal displacements.

This phenomenon can be attributed to the fundamental nature of phase correlation’s frequency-domain analysis. When inter-frame displacement is minimal, the resulting phase shifts become subtle and potentially ambiguous. For micro-textured surfaces with rich high-frequency content, this ambiguity is exacerbated as multiple frequency components contribute conflicting phase information, leading to unreliable peak detection in the inverse Fourier transform. The algorithm requires sufficient motion magnitude (typically 8% step size or greater) to generate distinctive frequency-domain signatures that overcome this ambiguity.

Template matching demonstrates even more extreme texture sensitivity, particularly evident in the catastrophic failure with compact windows on micro-textured surfaces (537.2 pixels at 2% step size versus 36.3 pixels on macro-texture). This order-of-magnitude degradation suggests that fine texture details create severe aliasing effects when the search window is insufficient to capture unique matching features. The narrow window of exceptional performance at 6% step size (4.0 pixels) on micro-textures, contrasted with selective excellence at 16%, 22%, and 24% on macro-textures, indicates that optimal correlation conditions are highly texture-specific.

### 5.2. Fundamental Limitations of Optical Flow

The complete unsuitability of optical flow for micro-textured surfaces represents a fundamental algorithmic limitation rather than a parameter tuning issue. The Lucas-Kanade formulation assumes brightness constancy and spatial smoothness—assumptions that are violated by high-frequency texture content. On micro-textured surfaces, mean errors of 95.9 pixels at 2% step size (compared to 5.1 pixels on macro-texture) confirm that the method cannot handle fine spatial details that change rapidly across neighboring pixels.

This finding has important theoretical implications. While optical flow performs marginally better on macro-textured surfaces at very low speeds, it still exhibits unacceptable failure rates (65–70%) even in its optimal regime. The catastrophic failure beyond 8% step size on all surface types confirms that optical flow is fundamentally unsuited to precision displacement measurement applications, regardless of texture characteristics or parameter optimization.

### 5.3. Window Size Effects and Computational Trade-Offs

The systematic comparison between 200 × 200 and 400 × 400 pixel windows reveals critical scaling relationships that extend beyond simple computational considerations. For phase correlation, larger windows provide approximately 2× error reduction while maintaining characteristic stability patterns. However, for template matching, window size effects are dramatically amplified on micro-textured surfaces, where insufficient window size leads to complete algorithmic breakdown.

The 4× computational overhead of larger windows is justified not merely by performance improvements but by fundamental reliability considerations. Template matching’s transition from 537.2 pixels mean error to 4.0 pixels at optimal conditions when moving from compact to standard windows on micro-textures exemplifies how window size can determine the difference between complete failure and exceptional performance.

### 5.4. Statistical Robustness and Reliability

The multi-run experimental design with randomized starting positions provides crucial insights into algorithm reliability. Phase correlation demonstrates excellent consistency in its stable operating range (std dev 1.05–1.61 pixels at optimal points), while template matching exhibits higher variability that increases dramatically on micro-textured surfaces. This variance analysis reveals that fine texture details create multiple local correlation maxima, increasing sensitivity to initialization and reducing tracking robustness.

The run-to-run consistency metrics have direct implications for sensor design. Applications requiring predictable performance across varying initialization conditions should prioritize phase correlation with appropriate window sizing, while template matching should be reserved for controlled environments where initialization can be carefully managed.

### 5.5. Practical Implementation Framework

Based on the comprehensive experimental evidence across both texture types, a hierarchical decision framework for algorithm selection emerges:

Primary texture classification: Determine whether the target surface exhibits macro or micro-texture characteristics. This fundamental distinction drives all subsequent algorithm selection decisions.

For macro-textured surfaces:

- General-purpose applications (8–26% step size): Phase correlation with 400 × 400 pixel windows provides consistent sub-3.5 pixel accuracy,
- Low-speed applications (2–6% step size): Optical flow with 200 × 200 windows offers marginal advantages but with high failure risk,
- Precision-calibrated systems: Template matching at 16%, 22%, or 24% step sizes achieves 2.2–2.8 pixel accuracy.

For micro-textured surfaces:

- Reliable tracking (8–30% step size): Phase correlation with 400 × 400 windows is the only consistently viable option (1.9–2.1 pixels),
- Precision requirements at 6% step size: Template matching with 400 × 400 windows can achieve 4.0 pixel accuracy but requires precise control,
- Optical flow should be categorically avoided regardless of operating conditions.

Critical avoidance zones:

- Phase correlation at 2–6% step sizes on all textures,
- Template matching with 200 × 200 windows on micro-textures,
- Optical flow for any precision application on micro-textures,
- All methods at step sizes exceeding 26% except phase correlation.

### 5.6. Implications for Sensor Design

These findings necessitate a fundamental reconsideration of optical displacement sensor design strategies. Rather than treating algorithm selection as a post-design optimization problem, the strong texture and parameter dependencies revealed by this study suggest that algorithm characteristics should drive sensor specifications from the outset.

The identification of texture-dependent performance patterns enables the development of adaptive tracking systems that can classify surface characteristics and automatically select appropriate algorithms and parameters. The quantitative performance boundaries established through this research provide the foundation for such intelligent sensor systems.

## 6. Conclusions

This comprehensive study has systematically evaluated three classical computer vision algorithms for optical displacement sensing across diverse surface textures and across diverse surface textures, window sizes, and step sizes. Through rigorous experimentation with multiple runs and randomized initializations, the research establishes quantitative performance boundaries that enable evidence-based algorithm selection for precision measurement applications.

The key findings demonstrate that surface texture characteristics fundamentally determine tracking algorithm performance. Phase correlation emerges as the most versatile method, achieving consistent sub-2 pixel accuracy in its stable operating range (8–30% step size) across both macro and micro-textured surfaces when using 400 × 400 pixel windows. However, its unexpected poor performance at very low step sizes (2–6%) challenges conventional assumptions about frequency-domain tracking methods.

Template matching exhibits extreme texture sensitivity, with performance ranging from exceptional accuracy at specific operating points (2.2 pixels at 16% on macro-texture, 4.0 pixels at 6% on micro-texture) to complete failure under non-optimal conditions. The method’s catastrophic failure with compact windows on micro-textured surfaces (537.2 pixels error) underscores the critical importance of proper window sizing.

Optical flow performs poorly under the restricted conditions tested here: fixed 2D planar surfaces undergoing pure translation with generalized micro- and macro-textures derived from metallographic images. In this laboratory-style setting, high-frequency texture content violates its underlying assumptions, leading to unacceptable error rates that preclude its use for precision metrology. We emphasize, however, that this conclusion applies to controlled displacement-sensing scenarios (e.g., CNC or microscope-based systems) and does not contradict the demonstrated effectiveness of optical-flow-based methods in other domains, such as aerial positioning or robotic navigation, where scene structure, motion dynamics, and imaging conditions differ fundamentally.

The research establishes that texture classification should precede algorithm selection in optical sensor design. For applications requiring robust performance across varying conditions, phase correlation with appropriate window sizing provides the optimal solution. Template matching should be reserved for specialized applications where both surface texture and motion parameters can be precisely controlled.

Overall, this work contributes a quantitative framework that replaces ad-hoc, trial-and-error tracker choice with systematic, evidence-based design principles for optical odometry and displacement sensing. These findings provide a scientific foundation for future sensor development and benchmarking. Future work should explore adaptive algorithms that can automatically classify texture characteristics and select optimal tracking methods, as well as investigate modern deep learning approaches within the same rigorous experimental framework. We acknowledge that the present study relies exclusively on synthesized image sequences, which—while enabling controlled benchmarking—inevitably idealize real-world imaging conditions. As a natural next step, future work will incorporate representative experimental image sets to validate and extend the simulation-based results.

These conclusions are directly shaped by the controlled experimental parameters—window size, step size, and surface texture—which define the operational boundaries of each method and ensure that the reported performance differences arise from algorithmic behaviour rather than uncontrolled variability.

## Figures and Tables

**Table 1:** Comparison of principal vision-based planar odometry approaches.

Approach	Use Cases	Strengths	Weaknesses
Phase correlation	Image registration, microscopy, blur-robust alignment	Illumination/offset invariance; efficient FFT refinement; GPU acceleration	Sensitive to low-texture regions; peak broadening under strong blur
Template matching	Visual tracking, PCB/industrial inspection	Simple formulation; scale-adaptive variants; FPGA acceleration	Template drift; poor generalisation under large appearance change
Optical flow	Dense motion estimation, robotics, high-speed vision	Dense field; strong CNN/transformer benchmarks; SAM-guided boundary precision	Brightness constancy assumption; weak on low-texture or occluded regions
Surface patterning	DIC, precision metrology, experimental mechanics	Optimised speckles; objective quality metrics; CNN assessors	Limited to controlled surfaces; risk of periodic mis-registration
Deep learning	FlowNet, RAFT, DeepDIC, hybrid physics-informed models	High accuracy; real-time inference; domain adaptation possible	Require large training data; limited traceability; weaker physical guarantees

**Table 2:** Controlled experimental parameters for performance evaluation.

Parameter	Values	Rationale/Reproducibility
Feed window size	200×200, 400×400 px	Balance between texture coverage and computational efficiency; identical regions used for all methods
Step size	2–30% of window dimension (2% increments)	Scale-invariant metric; ensures comparability across window sizes; fixed trajectory applied to all methods

**Table 3:** Safe operating zones (step-size ranges with ≤5 px mean error) for each algorithm, texture type, and window size. A dash (–) indicates no reliable operating zone was observed.

Texture Type	Window Size	Algorithm	Safe Op. Zone (% of Window)
Macro-texture	200×200	Phase Correlation	≥14%
		Template Matching	16%, 22%, 24% (isolated)
		Optical Flow	≤6% only
	400×400	Phase Correlation	8–30%
		Template Matching	16%, 22%, 24% (isolated)
		Optical Flow	≤6% only
Micro-texture	200×200	Phase Correlation	– (no consistent safe zone)
		Template Matching	≈6% only
		Optical Flow	–
	400×400	Phase Correlation	≥8%
		Template Matching	≈6% only
		Optical Flow	–

## Data Availability

The data presented in this study are available on request from the corresponding author. The data are not publicly available due to privacy restrictions.

## Abbreviations

The following abbreviations are used in this manuscript:

2D	Two-dimensional
3D	Three-dimensional
AI	Artificial intelligence
CLAHE	Contrast-limited adaptive histogram equalisation
CNN	Convolutional neural network
DIC	Digital image correlation
FFT	Fast Fourier transform
GPU	Graphics processing unit
NCC	Normalised cross-correlation
SNR	Signal-to-noise ratio

## References

[1] Blikharskyy Y., Kopiika N., Khmil R., Selejdak J., Blikharskyy Z. (2022). Review of Development and Application of Digital Image Correlation Method for Study of Stress–Strain State of RC Structures. Appl. Sci..

[2] Quan S., Liang X., Zhu H., Hirano M., Yamakawa Y. (2022). HiVTac: A High-Speed Vision-Based Tactile Sensor for Precise and Real-Time Force Reconstruction with Fewer Markers. Sensors.

[3] Mallya R., Uchil A.K., Shenoy S.B., Rai S.K., Shetty A. (2024). Application of Digital Image Correlation in Aerospace Engineering: Structural Health Monitoring of Aircraft Components. Aerosp. Sci. Technol..

[4] Mylo M.D., Poppinga S. (2024). Digital Image Correlation Techniques for Motion Analysis and Biomechanical Characterization of Plants. Front. Plant Sci..

[5] Dong Y., Pan B. (2017). A Review of Speckle Pattern Fabrication and Assessment for Digital Image Correlation. Exp. Mech..

[6] Lentz J., Sevil H.E., Fries D. (2025). Image Preprocessing to Enhance Phase Correlation of Featureless Images. Sci. Rep..

[7] Nasajpour-Esfahani N., Karimi S., Nasseri S., Borna H., Boostani A.F., Gao R., Huang W., Garmestani H., Liang S.Y. (2025). Advancements and Applications of Digital Image Correlation to Characterize Residual Stress: A Review. Mater. Charact..

[8] Rasmy L., Sebari I., Ettarid M. (2021). Automatic Sub-Pixel Co-Registration of Remote Sensing Images Using Phase Correlation and Harris Detector. Remote Sens..

[9] Foroosh H., Zerubia J., Berthod M. (2002). Extension of Phase Correlation to Subpixel Registration. IEEE Trans. Image Process..

[10] Wan X., Liu J.G., Yan H. (2015). The Illumination Robustness of Phase Correlation for Image Alignment. IEEE Trans. Geosci. Remote Sens..

[11] Xiong B., Zhang Q. (2022). On Quadratic Surface Fitting for Subpixel Motion Extraction from Video Images. Entropy.

[12] Guizar-Sicairos M., Thurman S., Fienup J. (2008). Efficient Subpixel Image-Registration Algorithms. Opt. Lett..

[13] Padfield D. (2012). Masked Object Registration in the Fourier Domain. IEEE Trans. Image Process..

[14] Li T., Wang J., Yao K. (2022). Subpixel Image Registration Algorithm Based on Pyramid Phase Correlation and Upsampling. Signal Image Video Process..

[15] Sharma S., Kulkarni R. (2023). State-Space Modeling Approach for Fringe Pattern Demodulation. Appl. Opt..

[16] Schubert F., Mikolajczyk K., Wilson R., Hancock E., Bors A., Smith W. (2013). Benchmarking GPU-Based Phase Correlation for Homography-Based Registration of Aerial Imagery. Computer Analysis of Images and Patterns, Proceedings of the CAIP 2013, York, UK, 27–29 August 2013.

[17] Li H., Tan B., Pandiyan V.P., Barathi V.A., Sabesan R., Schmetterer L., Ling T. (2025). Phase-Restoring Subpixel Image Registration: Enhancing Motion Detection Performance in Fourier-Domain Optical Coherence Tomography. J. Phys. D Appl. Phys..

[18] Ojansivu V., Rahtu E. (2007). Image Registration Using Blur-Invariant Phase Correlation. IEEE Trans. Pattern Anal. Mach. Intell..

[19] Chen Z., Chen Q., Chen W., Wang Y., Xu C., Li X., Dou Q. (2023). DPCN++: Differentiable Phase Correlation Network for Versatile Pose Registration. IEEE Trans. Pattern Anal. Mach. Intell..

[20] Annaby M., Fouda Y. (2024). Fast Template Matching and Object Detection Techniques Using *ϕ*-Correlation and Binary Circuits. Multimed. Tools Appl..

[21] Woodford O.J. (2018). Least Squares Normalized Cross Correlation. arXiv.

[22] Salaris M., Damiani A., Putti E., Stornaiuolo L. FPGA-Based Implementation of 2D Normalized Cross-Correlation for Large Scale Signals. Proceedings of the 2021 IEEE 6th International Forum on Research and Technology for Society and Industry (RTSI).

[23] Cheng J., Wu Y., AbdAlmageed W., Natarajan P. QATM: Quality-Aware Template Matching for Deep Learning. Proceedings of the IEEE/CVF Conference on Computer Vision and Pattern Recognition (CVPR).

[24] Gao B., Spratling M.W., Yao J., Xiao Y., You P., Sun G. (2022). Robust Template Matching via Hierarchical Convolutional Features from a Shape Biased CNN. Proceedings of the International Conference on Image, Vision and Intelligent Systems (ICIVIS 2021).

[25] Le M.-T., Tu C.-T., Guo S.-M., Lien J.-J.J. (2020). A PCB Alignment System Using RST Template Matching with CUDA on Embedded GPU Board. Sensors.

[26] Matthews I., Ishikawa T., Baker S. (2004). The Template-Update Problem. IEEE Trans. Pattern Anal. Mach. Intell..

[27] Gräßl C., Zinßer T., Niemann H. Illumination-Insensitive Template Matching with Hyperplanes. Proceedings of the DAGM Symposium.

[28] Alfarano A., Maiano L., Papa L., Amerini I. (2024). Estimating Optical Flow: A Comprehensive Review of the State of the Art. Comput. Vis. Image Underst..

[29] Winkler J.R. (2024). Error Analysis and Condition Estimation of the Pyramidal Form of the Lucas–Kanade Method in Optical Flow. Electronics.

[30] Xu H., Yang J., Cai J., Zhang J., Tong X. High-Resolution Optical Flow from 1D Attention and Correlation. Proceedings of the IEEE/CVF International Conference on Computer Vision (ICCV).

[31] Xu H., Zhang J., Cai J., Rezatofighi H., Tao D. GMFlow: Learning Optical Flow via Global Matching. Proceedings of the IEEE/CVF Conference on Computer Vision and Pattern Recognition (CVPR).

[32] Shi X., Huang Z., Li D., Zhang M., Cheung K.C., See S., Li H. Flowformer++: Masked Cost Volume Autoencoding for Pretraining Optical Flow Estimation. Proceedings of the IEEE/CVF Conference on Computer Vision and Pattern Recognition (CVPR).

[33] Zhou S., He R., Tan W., Yan B. SAMFlow: Eliminating Any Fragmentation in Optical Flow with Segment Anything Model. Proceedings of the AAAI Conference on Artificial Intelligence.

[34] Ishii I., Taniguchi T., Yamamoto K., Takaki T. (2010). 1000-fps Real-Time Optical Flow Detection System. Proc. SPIE.

[35] Baker S., Scharstein D., Lewis J.P., Roth S., Black M.J., Szeliski R. (2011). A Database and Evaluation Methodology for Optical Flow. Int. J. Comput. Vis..

[36] Chen Z., Shao X., Xu X., He X. (2018). Optimized Digital Speckle Patterns for Digital Image Correlation by Consideration of Both Accuracy and Efficiency. Appl. Opt..

[37] Wen Y., Wang J., Zheng L., Chen S., An H., Li L., Long Y. (2024). Method of Generating Speckle Patterns for Digital Image Correlation Based on Modified Conway’s Game of Life. Opt. Express.

[38] Hu X., Xie Z., Liu F. (2021). Assessment of Speckle Pattern Quality in Digital Image Correlation from the Perspective of Mean Bias Error. Measurement.

[39] Hu W., Sheng Z., Yan K., Miao H., Fu Y. (2021). A New Pattern Quality Assessment Criterion and Defocusing Degree Determination of Laser Speckle Correlation Method. Sensors.

[40] Kwon T.H., Park J., Jeong H., Park K. (2023). Assessment of Speckle-Pattern Quality Using Deep-Learning-Based CNN. Exp. Mech..

[41] Li Y., Xue Y., Tian L. (2018). Deep Speckle Correlation: A Deep Learning Approach toward Scalable Imaging through Scattering Media. Optica.

[42] Guelpa V., Laurent G.J., Sandoz P., Zea J.G., Clévy C. (2014). Subpixelic Measurement of Large 1D Displacements: Principle, Processing Algorithms, Performances and Software. Sensors.

[43] Dosovitskiy A., Fischer P., Ilg E., Häusser P., Hazırbaş C., Golkov V., van der Smagt P., Cremers D., Brox T. FlowNet: Learning Optical Flow with Convolutional Networks. Proceedings of the IEEE International Conference on Computer Vision (ICCV).

[44] Sun D., Yang X., Liu M.Y., Kautz J. PWC-Net: CNNs for Optical Flow Using Pyramid, Warping, and Cost Volume. Proceedings of the IEEE Conference on Computer Vision and Pattern Recognition (CVPR).

[45] Teed Z., Deng J. (2020). RAFT: Recurrent All-Pairs Field Transforms for Optical Flow. Proceedings of the European Conference on Computer Vision (ECCV).

[46] Yang R., Li Y., Zeng D., Guo P. (2022). Deep DIC: Deep Learning-Based Digital Image Correlation for End-to-End Displacement and Strain Measurement. J. Mater. Process. Technol..

[47] Duan X., Xu H., Dong R., Lin F., Huang J. (2023). Digital Image Correlation Based on Convolutional Neural Networks. Opt. Lasers Eng..

[48] Shan Y., Zhen M., Fill H.D. (2025). Research on Structural Mechanics Stress and Strain Prediction Models Combining Multi-Sensor Image Fusion and Deep Learning. Appl. Sci..

[49] Cheng X., Zhou S., Xing T., Zhu Y., Ma S. (2023). Solving Digital Image Correlation with Neural Networks Constrained by Strain–Displacement Relations. Opt. Express.

[50] Sjödahl M. (2019). Gradient Correlation Functions in Digital Image Correlation. Appl. Sci..

